# Medical College Notes

**Published:** 1894-07

**Authors:** 


### Medical College Notes.

THE Medico-Chirurgical College, of Philadelphia, has had its teaching faculty increased by the following appointments lately made by the board of trustees to various chairs in that institution: Dr. Isaac Ott, of Easton, Pa., professor of physiology; Dr. William E. Hughes, professor of clinical medicine; Dr. Albert E. Roussel, assistant professor of clinical and of practice of medicine; Dr. Charles W. Burr, clinical professor of nervous diseases; Dr. William C. Hollepeter, clinical professor of diseases of children and pediatrics; Dr. Arthur H. Cleveland, clinical professor of laryngology; Dr. Edward B. Gleason, clinical professor of otology, and Dr. William Blair Stewart, lecturer in therapeutics.

MR. WILLIAM DEERING, of Chicago, the harvest machine manufacturer, has given the Northwestern University $50,000, to found a new professorship in the medical school. This is a benefaction that is to be commended.

